# Genetic architecture constrains exploitation of siderophore cooperation in the bacterium *Burkholderia cenocepacia*

**DOI:** 10.1002/evl3.144

**Published:** 2019-10-02

**Authors:** Santosh Sathe, Anugraha Mathew, Kirsty Agnoli, Leo Eberl, Rolf Kümmerli

**Affiliations:** ^1^ Department of Plant and Microbial Biology University of Zürich Zürich Switzerland; ^2^ Department of Quantitative Biomedicine University of Zürich Zürich Switzerland

**Keywords:** Cheating, pleiotropy, genetic architecture, microbial cooperation, public goods, siderophores

## Abstract

Explaining how cooperation can persist in the presence of cheaters, exploiting the cooperative acts, is a challenge for evolutionary biology. Microbial systems have proved extremely useful to test evolutionary theory and identify mechanisms maintaining cooperation. One of the most widely studied system is the secretion and sharing of iron‐scavenging siderophores by *Pseudomonas* bacteria, with many insights gained from this system now being considered as hallmarks of bacterial cooperation. Here, we introduce siderophore secretion by the bacterium *Burkholderia cenocepacia* H111 as a novel parallel study system, and show that this system behaves differently. For ornibactin, the main siderophore of this species, we discovered a novel mechanism of how cheating can be prevented. Particularly, we found that secreted ornibactin cannot be exploited by ornibactin‐defective mutants because ornibactin receptor and synthesis genes are co‐expressed from the same operon, such that disruptive mutations in synthesis genes compromise receptor availability required for siderophore uptake and cheating. For pyochelin, the secondary siderophore of this species, we found that cheating was possible, but the relative success of cheaters was positive frequency dependent, thus diametrically opposite to the *Pseudomonas* and other microbial systems. Altogether, our results highlight that expanding our repertoire of microbial study systems leads to new discoveries and suggest that there is an enormous diversity of social interactions out there in nature, and we might have only looked at the tip of the iceberg so far.

**Impact Statement**

Cooperation is a widespread phenomenon observed in all domains of life including microbes, insects, animals, and humans. The evolutionary origin and maintenance of cooperation is a puzzle, because cooperative behaviors are costly to perform, yet they can generate benefits for other individuals than the cooperator. This means that cooperation can be undermined by selfish individuals (so‐called cheaters) who do not contribute to cooperation, but reap its benefits. Microbial systems are widely used to experimentally study this evolutionary puzzle. One of the most popular study system is the production of iron‐chelating siderophores by *Pseudomonas* bacteria, where secreted molecules are shared as public goods between cells. Here, we ask whether the findings from *Pseudomonas* are generalizable. We addressed this question by studying siderophore‐mediated social interactions in the opportunistic human pathogen *Burkholderia cenocepacia* H111, which produces two different siderophores: ornibactin and pyochelin. We found that both siderophores are secreted and shared among producers, but only pyochelin and not ornibactin could be exploited by cheating nonproducers. Molecular investigations revealed a novel way of how cheaters can be kept in check, a mechanism that genetically links siderophore synthesis and uptake, such that ornibactin non‐producers, which are potential cheaters, fail to produce receptors required for ornibactin uptake. In contrast, for pyochelin we found that nonproducers could act as cheaters, but only when common in the population, indicating that cheaters might be unable to invade from rare. Altogether, our results are markedly different from the *Pseudomonas* system and suggest that bacteria have evolved a large repertoire of mechanisms withstanding cheating and maintaining cooperation.

Siderophores are secondary metabolites secreted by bacteria to scavenge insoluble and host‐bound iron from the environment (Miethke and Marahiel 2007; Hider and Kong 2010). They are of major ecological importance, as they fulfill a wide range of functions. They constitute virulence factors in infections (Miethke and Marahiel 2007; Cassat and Skaar 2013), remediate heavy‐metal polluted environments (Braud et al. 2010; Hesse et al. 2018), and drive community dynamics by supressing the growth of competitors (Butaitė et al. 2017) or benefiting close relatives through cooperative molecule sharing (Griffin et al. 2004; Weigert and Kümmerli 2017). Especially the observation that the secretion of siderophores can constitute a cooperative act, benefiting individuals other than the producers, has attracted enormous attention (Griffin et al. 2004; Cordero et al. 2012; Julou et al. 2013; Andersen et al. 2015; Tekwa et al. 2017; Vasse et al. 2017; Granato et al. 2018). The key question in this context is how cooperative siderophore secretion can be evolutionarily maintained, given that siderophore‐negative cheater mutants can arise and freeride on the public goods produced by others (West et al. 2006; Özkaya et al. 2017). A large body of work has tackled this question and revealed that cheating and cheater control are major determinants of bacterial population dynamics in host infections, in laboratory experiments, and in environmental communities (Cordero et al. 2012; Andersen et al. 2015; Kümmerli et al. 2015; Bruce et al. 2017; Butaitė et al. 2017; O'Brien et al. 2017). However, despite the great advance these insights provide for our understanding of microbial social interactions and community dynamics, most of the work carried out so far stems from one type of siderophore (pyoverdine) produced by one type of bacterium (fluorescent pseudomonads, particularly *Pseudomonas aeruginosa*), but also see Cordero et al. (2012), Kümmerli et al. (2014), and Scholz and Greenberg (2015) for exceptions.

This limitation prompted us to test whether the findings reported for *P. aeruginosa* are generalizable, and also applicable to other bacterial systems. Thus, we set out to explore the social role of the two siderophores, ornibactin and pyochelin, produced by *Burkholderia cenocepacia* (Agnoli et al. 2006; Mathew et al. 2014; Mathew et al. 2016). Like *P. aeruginosa*, this bacterium is an opportunistic pathogen that can inhabit a wide range of environments (Coenye and Vandamme 2003; Sousa et al. 2011). Using this study system, we repeated a number of key experiments, which previously demonstrated the social nature of pyoverdine in *P. aeruginosa* (Ross‐Gillespie et al. 2007; Kümmerli et al. 2009; Ross‐Gillespie et al. 2009; Zhang and Rainey 2013). Specifically, we carried out siderophore secretion and supernatant cross‐use assays, and competed a siderophore‐negative mutant against producer strains across a range of conditions by manipulating competition time, culture mixing, strain density, and frequency. Although our results revealed that both siderophores of *B. cenocepacia* are secreted into the environment, we consistently found that only pyochelin, but not ornibactin, is an exploitable public good in this species.

This surprising finding motivated us to investigate the molecular basis of why ornibactin cannot be exploited in *B. cenocepacia*. We suspected that the genetic architecture of the siderophore locus could play a key role in determining whether nonproducers can exploit a specific siderophore. Specifically, we predict that cheating relies on the independent regulation of siderophore synthesis and receptor genes, which enables a strain deficient in siderophore production to express the receptor required for siderophore uptake. Such an independent regulation is indeed in place for pyoverdine in *P. aeruginosa* (Visca et al. 2007). For *B. cenocepacia*, the genes encoding the pyochelin synthesis machinery and the receptor are organized in different operons (Butt and Thomas 2017) and might thus also be regulatorily independent. Conversely, the ornibactin receptor gene is located downstream of the synthesis genes as part of the same operon (Agnoli et al. 2006; Butt and Thomas 2017), indicating strong positive regulatory linkage between siderophore synthesis and uptake. Here, we use a combination of gene expression analysis and strain engineering to test whether a deletion in a synthesis gene, which turns a producer into a nonproducer, negatively affects the expression of downstream receptor genes, and thus compromise the evolutionary success of nonproducers. Finally, we discuss whether such regulatory linkage could represent a robust mechanism to maintain cooperation or whether it can break across evolutionary time scales (Dos Santos et al. 2018).

## Methods

### BACTERIAL STRAINS

The experiments were performed with *Burkholderia cenocepacia* H111 (LMG 23991), a clinical isolate from a cystic fibrosis patient (Gotschlick et al. 2001). This isolate (hereafter referred to as wild type) produces two siderophores, ornibactin, and pyochelin (Darling et al. 1998; Sokol et al. 1999). This species further possesses a membrane‐embedded siderophore‐independent iron uptake mechanism (Mathew et al. 2014), which we did not focus on in this study, because its contribution to iron uptake is relatively minor under the culturing conditions used. We further used in‐frame deletion mutants defective for either the production of ornibactin (H111∆*orbJ*), pyochelin (H1111∆*pchAB*), or both ornibactin and pyochelin (H111∆*orbJ*∆*pchAB*) (see Mathew et al. 2014). For competition assays, we chromosomally tagged the siderophore nonproducer strain (H111∆*orbJ*∆*pchAB*) with a constitutive fluorescent mCherry marker, inserted at the attTn7 site using a tri‐parental mating (Choi and Schweizer 2006). The donor and helper strains used for conjugation are listed in table S1. We checked whether *mcherry* gene insertion had any adverse effect by comparing the growth of H111∆*orbJ*∆*pchAB* and H111∆*orbJ*∆*pchAB‐mcherry* as monocultures and in competition with each other both in iron‐rich and iron‐poor media (see below). All the strains were maintained as clonal populations in 25% glycerol stocks at −80°C.

### MEDIA AND CULTURING CONDITIONS

Prior to all growth assays, we inoculated strains from −80°C glycerol stocks in 50‐mL sterile falcon tubes containing 10 mL Lysogeny broth (LB), and incubated them for approximately 15 hours at 37°C, shaken at 220 RPM. The overnight cultures were pelleted using a centrifuge (5000 RPM, 22°C, 3 minutes), aseptically washed and resuspended twice with 0.8% sterile NaCl solution, and adjusted to OD_600nm_ = 1. For all our experiments, we used casamino acids medium (CAA: 5 g/L casamino acids, 1.18 g/L K_2_HPO_4_ × 3H_2_O, 0.25 g/L MgSO_4_ × 7H_2_O, 25 mM HEPES). The CAA was supplemented with either 100 µM 2,2′‐bipyridine, a strong iron chelator inducing iron starvation (henceforth, iron‐poor), or 100 µM FeCl_3_ to make the medium iron replete (henceforth, iron rich). The concentrations of 2,2′‐bipyridine and FeCl_3_ were chosen based on the outcome of an initial growth experiment, where we subjected the wild‐type and the mutant strains to a range of 2,2′‐bipyridine and FeCl_3_ concentrations. This experiment was performed in a 96‐well plate, where bacterial strains were inoculated in 200 µL CAA medium at the starting density of OD_600nm_ = 1 × 10^−4^. The plates were incubated at 37°C in a microplate reader (SpectraMax Plus 384; Molecular Devices, USA) and the growth was monitored by measuring OD at 600 nm every 15 minutes for 24 hours. The plates were shaken for 10 seconds prior to measuring OD. This preliminary experiment revealed that the growth of H111∆*orbJ*∆*pchAB* was significantly compromised with 100 µM 2,2′‐bipyridine, whereas the wild‐type and the two single siderophore mutants could still grow well (Fig. S1).

### SIDEROPHORE DETECTION BY CAS ASSAY

We used the chrome azurol S (CAS) assay (Schwyn and Neilands 1987) to quantify the amount of siderophores secreted by *B. cenocepacia* into the extracellular medium. We grew all strains in 10 mL iron‐poor CAA in a shaken incubator at 37°C, 220 RPM. After 24 hours, the cell cultures were centrifuged (7000 RPM, 5 minutes, and 22°C), the supernatants collected and filtered through 0.2 µm sterile Whatman filters (GE Healthcare, Switzerland). We then mixed 0.5 mL of 20 times diluted supernatant with 0.5 mL of freshly prepared CAS reagents. This mixture was incubated in the dark for 30 minutes and the blue to orange color change, which is proportional to the amount of siderophores present in supernatant, was measured through absorbance at 630 nm using a spectrophotometer (Ultrospec 2100 pro; Amersham Biosciences, UK). We used the CAA medium as the negative control and calculated the siderophores activity relative to the wild type.

### SUPERNATANT ASSAY

To test whether the siderophore nonproducing mutant (H111∆*orbJ*∆*pchAB*) can access secreted ornibactin and pyochelin, we grew this strain in the presence of supernatants from donor strains harvested from iron‐poor and iron‐rich media. For iron‐poor conditions, the supernatants from the wild type contains both ornibactin and pyochelin, whereas the supernatants of H111∆*orbJ* or H111∆*pchAB* only contain pyochelin or ornibactin, respectively. For iron‐rich conditions and for H111∆*orbJ*∆*pchAB*, supernatants should not contain any siderophores. To generate supernatants, we grew donor strains in 50 mL falcon tubes containing either 10 mL iron‐rich or iron‐poor CAA. The tubes were incubated at 37°C with shaking (220 RPM) for 24 hours, after which we centrifuged the grown cultures at 5,000 RPM for 3 minutes at room temperature, and filter sterilized the supernatants with 0.2 µm filters. We then grew the siderophore‐negative mutant (H111∆*orbJ*∆*pchAB*) in a medium containing 70% CAA supplemented with 30% supernatant. We opted for a full‐factorial design, where the growth effects of supernatants from both iron‐poor and iron‐rich conditions on H111∆*orbJ*∆*pchAB* were examined in both iron‐poor and iron‐rich media. We performed the experiments in 96‐well plates, with a starting inoculum of OD_600nm_ = 0.01 and static incubation at 37°C, and growth measurements of the double knockout at OD_600nm_ after 24 hours.

### COMPETITION ASSAYS

We conducted competition assays between the siderophore nonproducer (H111∆*orbJ*∆*pchAB*‐*mcherry*) and the three siderophore producers (wild type, H111∆*orbJ*, H111∆*pchAB*) to investigate whether the siderophore nonproducer can act as a cheater, outcompeting the siderophore‐producing cooperators in cocultures. The competition assays were performed in sterile, flat bottom 24 well plates (Falcon) containing 1.5 mL iron‐rich or iron‐poor CAA. We always grew monocultures alongside with mixed cultures under identical conditions. Because the relative success of a putative cheater strain can vary in response to environmental conditions, we manipulated four important variables in our competition assays: (1) time: we assessed the relative success of competing strains after 24 hours and 48 hours; (2) culturing condition: we carried out competition assays under static and shaken (170 RPM) conditions; (3) cell density: mixed cultures were initiated at four different starting densities, OD_600nm_ = 1 × 10^−4^, 1 × 10^−3^, 1 × 10^−2^, or 1 × 10^−1^, and (4) strain frequency: strain volumetric mixing ratio was varied from 1:99, 10:90; 25:75, 50:50, 75:25, 90:10, to 99:1. If not indicated otherwise, then the standard culturing condition included a 1:1 strain mix, starting OD_600nm_ = 0.01, incubated at 37°C in a static incubator. All competition experiments were performed in at least 15‐fold replication. We used a flow cytometer to determine the ratio of the two competitors before and after the competition. Thanks to the mCherry‐tag, H111∆*orbJ*∆*pchAB‐mcherry* could unambiguously be distinguished from its competitor (Fig. S2). All flow cytometric analyses were conducted on a LSRFortessa flow cytometer (BD Biosciences, USA), where mCherry was excited at 561 nm and fluorescence emission was quantified with a 610/20 nm bandpass filter. For every replicate, 100,000 bacterial events were recorded and both the initial and the final ratio of two strains in a mix was calculated. We used monocultures of H111∆*orbJ*∆*pchAB‐mcherry* to estimate the frequency of cells that were mCherry negative (e.g., 0.05% in the case shown in Fig. S2b), and then adjusted the strain frequencies in mixes accordingly. The flow cytometry data were analyzed using the software package FlowJo (TreeStar). The relative fitness of the siderophore‐deficient strain was calculated as: *v* = [*a*_t_(1 − *a*_0_)]**/**[*a*_0_(1 − *a*_t_)], where *a*_0_ and *a*_t_ are its initial and the final frequencies in the mixed population, respectively (Ross‐Gillespie et al. 2007). We log‐transformed *v*‐values, whereby *v* < 0 indicates a decrease and *v* > 0 an increase in the relative fitness of H111∆*orbJ*∆*pchAB‐mcherry* compared to its competitor.

### GENE EXPRESSION ANALYSIS

We used quantitative real‐time PCR (qPCR) to measure mRNA levels of the following three genes of the ornibactin locus encoding: (1) the nonribosomal peptide synthetase *orbI* involved in ornibactin biosynthesis, (2) the ferriornibactin receptor *orbA*, and (3) the housekeeping gene *recA* as a control. Figure 4A shows where these genes are located within the siderophore cluster and Table S2 lists the primers used for cDNA amplification. *B. cenocepacia* strains were grown in 250 mL Erlenmeyer flasks containing 50 mL sterile iron‐poor and iron‐rich CAA (37°C, shaken) until the cultures reached mid‐exponential phase (OD_600nm_ = 0.5 in iron‐rich media after 6 hours of inoculation, and OD_600nm_ = 0.2–0.5 in iron‐poor media after 10 hours of inoculation). RNA was isolated and purified from three independent cultures as described elsewhere (Pessi et al. 2007; Lardi et al. 2015). Briefly, 45 mL culture was mixed with 5 mL of 10% cold tris‐HCL saturated phenol (pH 8.0) and the cells were harvested using a centrifuge at 4°C, 7000 RPM for 5 minutes. The cell pellets were immediately flash frozen using liquid nitrogen and the frozen cell pellets were stored at −80°C. RNA was isolated using a hot acid phenol‐chloroform extraction method (Pessi et al. 2007). The total RNA was purified using RNeasy purification kit (Qiagen) and the contaminating genomic DNA was removed by two rounds of DNase I treatment (Promega I). Once the absence of genomic DNA was confirmed (with a 40‐cycle PCR and a primer amplifying H111 genomic DNA; Table S2) the RNA was once again purified using RNeasy kit, and its quality checked with NanoDrop (ThermoFisher Scientific). First‐strand cDNA was synthesized using 5 μg pure RNA and M‐MLV RT (Promega). We performed qPCR using pure cDNA and a specific qPCR kit (Agilent Technologies). The expression levels for a given gene in mutants was compared after normalizing its levels to the wild‐type levels.

### CONSTRUCTION OF A STRAIN CONSTITUTIVELY EXPRESSING THE ORNIBACTIN RECEPTOR *orbA*

To experimentally express the *orbA* receptor gene in the H111∆*orbJ*∆*pchAB* background, we cloned an approximately 1 kb *orbA* gene fragment into the modified expression plasmid pBBR1MCS5‐Tp (Kovach et al. 1995). The recombinant plasmid, from which *orbA* was constitutively expressed from the lac promoter, was then transformed into *E. coli* TOP 10, and subsequently transferred to H111∆*orbJ*∆*pchAB* by conjugation (Choi and Schweizer 2006). The recombinant colonies were selected on *Pseudomonas* isolation agar (*Burkholderia* can grow on this medium) supplemented with trimethoprim (100 μg/mL) and verified by PCR as well as sequencing. We found that the overexpression of *orbA* in H111∆*orbJ*∆*pchAB* (H111∆*orbJ*∆*pchAB:orbA*) mildly but significantly affected its growth and the relative fitness compared to its parental strain H111∆*orbJ*∆*pchAB* (Fig. S3), indicating that plasmid carriage is costly. We repeated the supernatant and the competition assays described above using H111∆*orbJ*∆*pchAB:orbA* to test whether this strain can cheat on siderophore producers (by taking the plasmid carriage cost into account).

### STATISTICAL ANALYSIS

All statistical analyses were performed with R 2.8.0 (http://www.r-project.org). We used linear models (LM) to test whether strains produce significantly different amounts of siderophores and whether the growth of H111∆*orbJ*∆*pchAB* is affected by the source of the supernatant. For competition assays, we used one‐sample *t*‐tests to test whether the relative fitness of H111∆*orbJ*∆*pchAB* significantly differs from *v* = 0. We further used LM to test whether the manipulated factors (time, culturing conditions, strain density, and frequency) affected the relative fitness of H111∆*orbJ*∆*pchAB*. In the cases of multiple comparisons, we adjusted the *P*‐values using the Tukey's HSD method (Montgomery 2017).

## Results

### *Burkholderia cenocepacia* H111 SECRETES ORNIBACTIN AND PYOCHELIN INTO THE MEDIA

**Figure 1:**
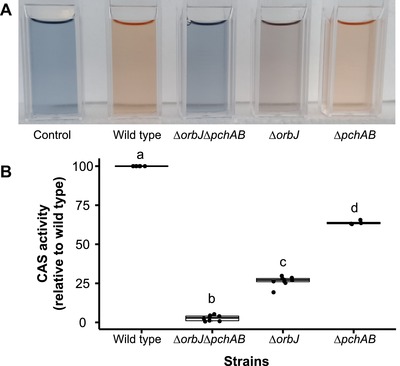
*Burkholderia cenocepacia* H111 secretes ornibactin and pyochelin into the media. (A) The colorimetric CAS assay detects siderophore activity in the extracellular cell‐free supernatant by inducing a color change from blue to orange. (B) The quantification of this color change through absorbance at 630 nm shows no siderophore activity in the CAA medium and for the double mutant (H111∆*orbJ*∆*pchAB*), which is unable to produce ornibactin and pyochelin. The supernatant of the wild type H111 showed highest siderophore activity, followed by the ornibactin producer (H111∆*pchAB*) and the pyochelin producer (H111∆*orbJ*). Different letters above the boxes indicate statistically significant differences in CAS activities measured as absorbance at 630 nm (one‐way ANOVA: *F*_3,24_ = 3482; *P* < 0.0001; the *P*‐values were corrected for multiple comparisons using Tukey's HSD).

We first used the colorimetric CAS assay to test whether *B. cenocepacia* H111 secretes its siderophores ornibactin and pyochelin into the extracellular medium, thus potentially generating a public good (Fig. 1A). We observed significant CAS activities in all producer strains, although activities differed between them (analysis of variance [ANOVA]: *F*_3,24_ = 3482; *P* < 0.0001, Fig. 1B). CAS activity was highest for the wild type supernatant followed by a stepwise decline from H111∆*pchAB* (ornibactin producer) to H111∆*orbJ* (pyochelin producer). The higher CAS activity for H111∆*pchAB* is probably due to ornibactin having a higher iron affinity than pyochelin (Thomas 2007). As expected, the supernatant of the siderophore‐deficient strain H111∆*orbJ*∆*pchAB* showed almost completely abrogated CAS activity.

### NONPRODUCERS CAN FREELY USE SECRETED PYOCHELIN, WHEREAS ACCESS TO ORNIBACTIN IS RESTRICTED

**Figure 2:**
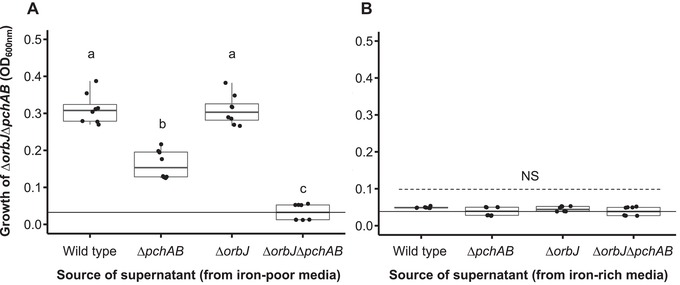
The siderophore nonproducer H111∆*orbJ*∆*pchAB* can take up supplemented siderophores, but growth induction differs between siderophore types. We tested whether the siderophore nonproducer H111∆*orbJ*∆*pchAB* can benefit from exogenous siderophores present in the supernatant of donor strains. (A) Supernatants from siderophore producers grown for 24 hours in iron‐poor media significantly stimulated the growth of the siderophore nonproducer H111∆*orbJ*∆*pchAB*. However, growth stimulation was reduced with supernatants from the H111∆*pchAB* donor strain containing only ornibactin. (B) In our control experiments, the growth‐stimulatory effects disappeared when supernatants were harvested from iron‐rich media, which contain little or no siderophores. Different letters above the boxes indicate statistically significant differences between treatments (one‐way ANOVA with *n* = 8 per treatment).

We found that the growth of the siderophore nonproducer H111∆*orbJ*∆*pchAB* was stimulated in supernatants containing siderophores from donor strains (Fig. 2A), demonstrating that siderophores can be shared between cells. However, there were significant donor effects with regard to the level of growth stimulation (ANOVA: *F*_4,27_ = 96.5; *P* < 0.0001). Although nonproducer cells grew equally well in the supernatants from the wild type and H111∆*orbJ*, stimulation was significantly compromised when grown in the supernatant of H111∆*pchAB* (Tukey's HSD: *t*_27_ = 8.76; *P* < 0.0001). This result was a first indication that ornibactin might be less accessible to nonproducers than pyochelin. Our control experiments confirmed that the observed growth stimulation was directly driven by siderophores, because all effects disappeared when H111∆*orbJ*∆*pchAB* grew with supernatants lacking siderophores (Fig. 2B, ANOVA: *F*_3,28_ = 2.56; *P* = 0.0748) or in iron‐rich medium, where siderophores are not required (Fig. S4).

### COMPETITION ASSAYS SUGGEST THAT ONLY PYOCHELIN BUT NOT ORNIBACTIN IS AN EXPLOITABLE SIDEROPHORE

**Figure 3:**
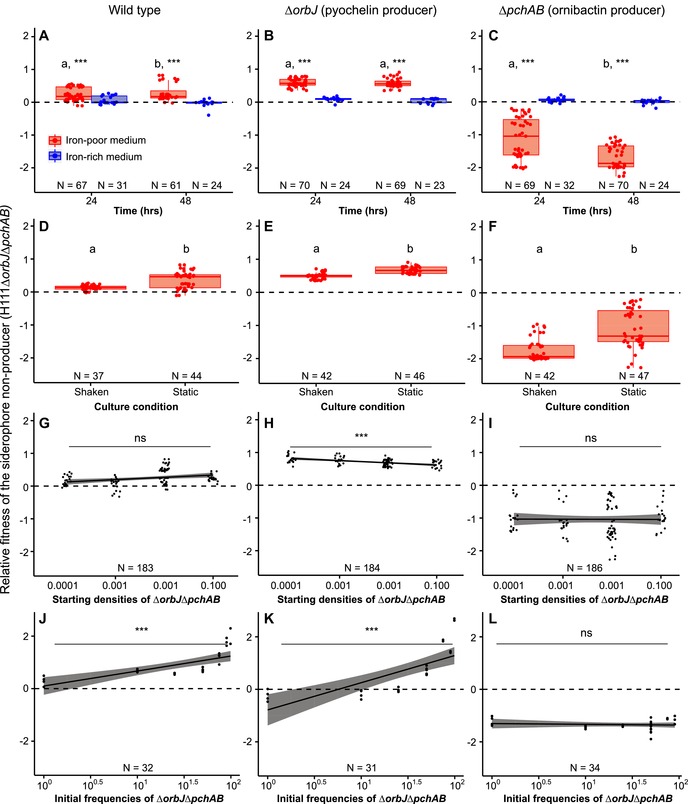
The siderophore nonproducer H111∆*orbJ*∆*pchAB* can cheat on the wild type and the pyochelin producer, but not on the ornibactin producer. We competed the siderophore nonproducer H111∆*orbJ*∆*pchAB* tagged with a constitutive mCherry marker against the wild type (first column), the pyochelin producer H111∆*orbJ* (second column), and the ornibactin producer H111∆*pchAB* (third column) across a range of culturing conditions. All plots show the relative fitness of the siderophore nonproducer, and the dashed horizontal lines represent fitness parity (i.e., when none of the two strains wins the competition). (A–C) Summary of relative fitness values across all conditions, showing that the siderophore nonproducer acts as cheater in competition against the wild type and the pyochelin producer, but loses against the ornibactin producer under iron‐poor conditions. Under iron‐rich conditions, there is fitness parity between strains, confirming that the observed fitness patterns under iron limitation are mediated by siderophores. (D–F) Relative fitness values of the siderophore nonproducer under shaken versus static culturing conditions. (G–I) Relative fitness values of the siderophore nonproducer across a range of starting cell densities. (J–L) Relative fitness values of the siderophore nonproducer across a range of strain mixing frequencies. ANOVAs (A–F) and linear regression analysis (G–L) were used to compare the relative fitness of nonproducers across the different culturing conditions. Different letters indicate statistically significant differences between treatments. Asterisks in (A–C) indicate cases where the relative fitness of the nonproducer is different from the expected *v* = 0; *t*‐tests, *P* < 0.001, whereas asterisks in (G–L) indicate significant regression effects of cell density and frequency. The sample sizes (*N*) for each treatment are indicated above the abscissas.

An important feature of microbial cheating is that public good nonproducers perform poorly in monoculture, but can outcompete producers in mixed culture, where they capitalize on the public goods produced by others (West et al. 2006). To test these predictions, we first grew the four strains as monocultures in CAA medium supplemented with either iron or a concentration gradient of the synthetic iron chelator 2,2′‐bipyridine. We found that the growth of the siderophore nonproducer (H111∆*orbJ*∆*pchAB*) became significantly compromised compared to the siderophore producers as soon as the iron chelator was added (Fig. S1). Next, we cocultured the siderophore nonproducer together with the siderophore producers (wild type, H111∆*orbJ* or H111∆*pchAB*) across a range of culturing conditions (Fig. 3). We consistently observed that the nonproducer outcompeted the wild type (ANOVA: *F*_1,120_ = 36.9; *P* < 0.0001) and the pyochelin producer (H111∆*orbJ*; *F*_1,119_ = 471.3; *P* < 0.0001) under iron limitation (Fig. 3), and thus acted as a cheater. In stark contrast, the nonproducer was unable to cheat on the ornibactin producer (H111∆*pchAB*) and lost under all conditions (Fig. 3; *F*_1,128_ = 235.5; *P* < 0.0001). In iron‐rich media, where siderophores are neither produced nor needed, the fitness differences between the siderophore producers and the nonproducer disappeared (Fig. 3A–C).

### CULTURING CONDITIONS AFFECT COMPETITION, BUT OFTEN DIFFERENTLY AS COMPARED TO THE *P. aeruginosa* PYOVERDINE SYSTEM

Work on pyoverdine sharing in *P. aeruginosa* showed that longer culturing times can lead to more extreme fitness outcomes (Kümmerli et al. 2009). We found this to hold true in two of our three competition experiments (Fig. 3A–C). In comparison to an experimental time course of 24 hours, the relative fitness of the nonproducer after 48 hours was significantly higher in competition with the wild type (Fig. 3A; ANOVA: *F*_1,42_ = 4.57; *P* = 0.0382), unchanged in competition with the pyochelin producer H111∆*orbJ* (Fig. 3B; *F*_1,41_ = 0.002; *P* = 0.9578), and significantly lower in competition with the ornibactin producer H111∆*pchAB* (Fig. 3C; *F*_1,45_ = 36.58; *P* < 0.0001).

Shaking is supposed to increase the mixing of cells and public goods, and has been shown to improve pyoverdine cheating abilities in *P. aeruginosa* (Leinweber et al. 2017). We found the opposite to be the case for the *Burkholderia* siderophores (Fig. 3D–F). Relative fitness of the nonproducer was consistently higher in static compared to shaken cultures (ANOVAs: Fig. 3D, against wild type: *F*_3,78_ = 12.65; *P* < 0.0001; Fig. 3E, against H111∆*orbJ*: *F*_3,85_ = 21.71; *P* < 0.0001; Fig. 3F; against H111∆*pchAB*: *F*_3,86_ = 37.38; *P* < 0.0001).

Previous work indicated that nonproducers can exploit siderophores under static conditions more efficiently at high cell density (Ross‐Gillespie et al. 2007; Scholz and Greenberg 2015). We found that cell density at inoculation had no or only a minor effect on the relative fitness of the nonproducer (regression analyses: Fig. 3G, against wild type: *F*_1,100_ = 0.43, *P* = 0.5098; Fig. 3H, against H111∆*orbJ*: *F*_1,97_ = 11.69, *P* = 0.0009; Fig. 3I, against H111∆*pchAB*: *F*_1,100_ = 0.69, *P* = 0.4054).

Finally, it was reported that *P. aeruginosa* pyoverdine nonproducers were more successful at outcompeting producers when rare (Ross‐Gillespie et al. 2007). When probing for this relationship in our *Burkholderia* system, we observed the opposite pattern in two out of three cases: nonproducers experienced significantly higher relative fitness advantages when more common in competition against the wild type (regression analysis: Fig. 3J; *F*_1,30_ = 46.2, *P* < 0.0001) and the pyochelin producer H111∆*orbJ* (Fig. 3K; *F*_1,29_ = 226.6, *P* < 0.0001). In the latter case, the nonproducer even lost the competition at initial frequencies smaller than 10%, indicating that pyochelin nonproducers might not be able to invade from rare. In competition with the ornibactin producer H111∆*pchAB*, the nonproducer had severe fitness disadvantages at all the frequencies (Fig. 3L; *F*_1,29_ = 1.24, *P* = 0.2730).

### DELETION OF THE *orbJ* ORNIBACTIN SYNTHESIS GENE REDUCES mRNA LEVELS OF THE DOWNSTREAM RECEPTOR GENE

**Figure 4:**
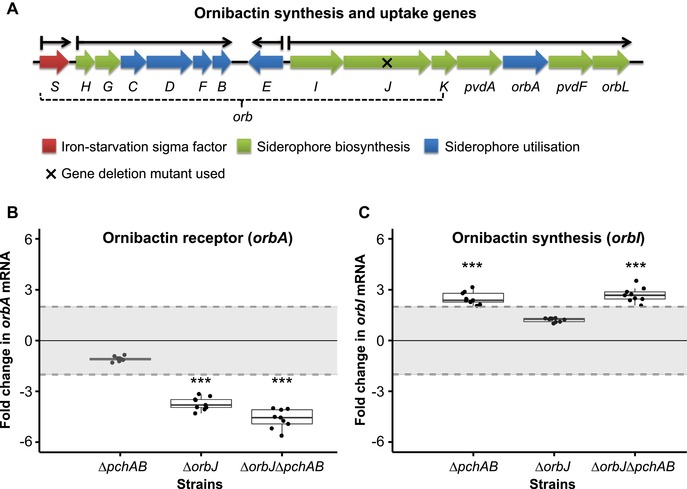
Genetic architecture and the expression of ornibactin genes. (A) The ornibactin synthesis and utilization genes are organized in three clusters (*orbIJK‐pvdA*‐*orbA*‐*pvdF‐orbL*, orbE, *orbHGCDFB*). *orbA* and *orbJ* encode the ferriornibactin receptor and the nonribosomal peptide synthetase, respectively. The details of gene organization and regulation can be found in Agnoli et al. (2006), Thomas (2007), and Butt and Thomas (2017). We used qPCR to quantify changes in mRNA levels in the ornibactin synthesis (*orbI*) and receptor gene (*orbA*) in all siderophore mutants relative to the wild type. We asked whether the genetic architecture of the siderophore locus affects mRNA levels in mutants lacking the siderophore synthesis gene *orbJ*. (B) Fold‐change in mRNA levels of *orbA*, the ornibactin receptor gene located downstream of the mutated *orbJ* gene. (C) Fold‐change in mRNA levels of *orbI*, an ornibactin synthesis gene located upstream of the mutated *orbJ* gene. All values are scaled relative to the mRNA levels in the wild type. Dashed lines and shaded areas depict the interval [−2|+2], in which mRNA level changes were not considered biologically significant; asterisks indicate significantly different gene expression levels compared to the wild type (*P* < 0.001), based on one‐sample *t*‐tests (*n* = 9).

The ornibactin synthesis gene *orbJ* and the receptor encoding gene *orbA* are part of the same operon. Therefore, we hypothesized that mutations in *orbJ* might negatively affect the expression of the downstream *orbA* gene (Fig. 4A). Such negative effects could include lower *orbA* expression levels or altered mRNA stability, which would in turn curb receptor synthesis and cheating opportunities. Compatible with our hypothesis, we found that *orbA* mRNA levels were significantly reduced in H111∆*orbJ* and H111∆*orbJ*∆*pchAB*, the two strains lacking the upstream *orbJ* gene (one‐sample *t*‐tests relative to the wild type; H111∆*orbJ*: *t*_8_ = −21.38, *P* < 0.0001; H111∆*orbJ*∆*pchAB*: *t*_8_ = −19.62, *P* < 0.0001; Fig. 4B). Conversely, *orbA* mRNA levels remained unchanged in H111∆*pchAB*, which possesses an intact *orbJ* (*t*_8_ = 1.71, *P* = 0.1250). If our hypothesis is correct, negative effects caused by *orbJ* mutations should only affect downstream, but not upstream genes. In support of this, we found that mRNA levels were not negatively affected in the upstream gene *orbI*, and even slightly increased in H111∆*orbJ* (one‐sample *t*‐tests: *t*_8_ = 5.34, *P* < 0.001) and H111∆*orbJ*∆*pchAB* (*t*_8_ = 11.63, *P* < 0.0001; Fig. 4C).

### OVEREXPRESSION OF *orbA* FROM PLASMIDS ENABLES H111∆*orbJ*∆*pchAB* TO CHEAT ON ORNIBACTIN PRODUCERS

**Figure 5:**
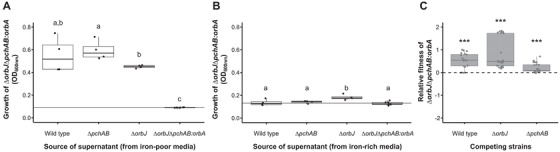
Overexpression of the ornibactin receptor gene from a plasmid restores ornibactin growth stimulation in the siderophore nonproducer and enables efficient cheating on the wild type. We repeated the supernatant assay described in Figure 2, but this time, we supplemented the supernatants from the siderophore producers to H111∆*orbJ*∆*pchAB:orbA*, overexpressing the receptor gene *orbA* from a plasmid. (A) The supernatant from H111∆*pchAB*, containing only ornibactin, now stimulated the growth of the siderophore non‐producer to a similar levels as the wild type, and even more so than the supernatant from H111∆*orbJ*, containing only pyochelin. This demonstrates that the nonproducer was no longer short of ornibactin receptors. (B) In our control experiments, the growth‐stimulatory effects disappeared when supernatants were harvested from iron‐rich media that contain little or no siderophores. Different letters above the boxes in (A) and (B) indicate statistically significant differences between treatments based on one‐way ANOVAs with *n* = 24. (C) We repeated the competition experiment described in Figure 3A–C, but this time, we competed the siderophore producers against H111∆*orbJ*∆*pchAB:orbA*, overexpressing the receptor gene *orbA* from a plasmid. We found that this nonproducer could significantly outcompete all siderophore producers, including H111∆*pchAB*, producing only ornibactin (relative fitness compared to the expected *v* = 0 using one sample *t*‐tests with *n* = 24; ***P < 0.0001 for all comparisons). The dashed horizontal line represents fitness parity (i.e., when none of the two strains wins the competition). Because the relative fitness values did not significantly differ between static and shaken conditions, the two treatments were merged.

To validate that the inability of H111∆*orbJ*∆*pchAB* to cheat on ornibactin producers is due to reduced receptor availability, we introduced a plasmid with a constitutively expressed *orbA* receptor gene into H111∆*orbJ*∆*pchAB*, thereby bypassing the genetic linkage in the operon. We first repeated the supernatant assay shown in Figure 2, but this time fed supernatants containing siderophores to the overexpresser H111∆*orbJ*∆*pchAB:orbA*. We found that this mutant was now equally well stimulated by the supernatants from the wild type and H111∆*pchAB* (producing only ornibactin), suggesting that ornibactin uptake is no longer constrained (Fig. 5A). Our control experiments confirmed again that the growth stimulatory patterns are driven by siderophores, as the observed effects disappeared when H111∆*orbJ*∆*pchAB:orbA* was either grown with supernatants from iron‐rich media containing little siderophores (Fig. 5B), or with supernatants containing siderophores but replenished with iron (Fig. S5).

We then repeated the competition assays using H111∆*orbJ*∆*pchAB:orbA* as the potential cheater strain (Fig. 5C). Similar to our previous finding (Fig. 3), we found that this siderophore negative mutant could act as cheater and outcompete the wild type (one‐sample *t*‐test: *t*_23_ = 7.84, *P* < 0.0001) and the pyochelin producer H111∆*orbJ* (*t*_23_ = 5.93, *P* < 0.0001). However, in stark contrast to our previous results (Fig. 3), we observed that the *orbA* overexpresser could also successfully cheat and outcompete the ornibactin producer H111∆*pchAB* (*t*_23_ = 4.80, *P* < 0.0001), indicating that insufficient receptor availability is indeed the cause that constrains cheating in H111∆*orbJ*∆*pchAB*.

## Discussion

There is a comprehensible tendency in biology to extrapolate from work on model organisms to the large diversity of nonmodal systems existing in nature (Levy and Currie 2014). In this context, research on the production and sharing of pyoverdine by the opportunistic human pathogen *P. aeruginosa* has become a prime example of cooperation in bacteria. Findings from this study system—including the observation that pyoverdine‐deficient mutants act as cheaters, potentially bringing cooperation to collapse (Griffin et al. 2004), and that cheaters perform best in shaken cultures (Leinweber et al. 2017), at high cell density (Ross‐Gillespie et al. 2009) and when rare (Ross‐Gillespie et al. 2007)—have often been interpreted as general patterns applicable to other microbial cooperative systems (West et al. 2006; Zhou et al. 2014; Bruger and Waters 2015; Özkaya et al. 2017). Here, we studied siderophore production in *B. cenocepacia* H111 and found that patterns of siderophore sharing and exploitation can be very different from the ones reported for *P. aeruginosa*. Most importantly, we observed that secreted ornibactin (the primary siderophore of this species) cannot be exploited by nonproducers, because the genetic architecture of the ornibactin locus implies that a deficiency in siderophore production leads to the concomitant downregulation of ornibactin receptor production. Moreover, we found that pyochelin (the secondary siderophore of *B. cenocepacia* H111) can be efficiently exploited by nonproducers, but the relative success of cheaters was independent of cell density, and highest in static cultures and when wild‐type *B. cenocepacia* was rare, thus opposite to the patterns observed for pyoverdine in *P. aeruginosa*. Our findings highlight that we have to be careful with extrapolations, but rather embrace the diversity offered by nature, which can lead, as in our case, to new discoveries and offer a more complete picture of the diversity of social interactions in microbes.

We have discovered a novel mechanism that secures benefits of secreted siderophores to producers and limits the ability of nonproducers to access public goods. This mechanism entails tight linkage between the ornibactin synthesis and receptor genes as part of the same operon. A consequence of this linkage is that a deletion in the synthesis gene negatively affects the downstream receptor gene, such that ornibactin nonproducers are short of receptors for ornibactin uptake. Reduced receptor availability is best demonstrated by our supernatant feeding assays showing that the siderophore nonproducer H111∆*orbJ*∆*pchAB* can still take up ornibactin to some extent (Fig. 2A), but growth stimulation was much reduced compared to pyochelin and relative to our engineered strain overexpressing the ornibactin receptor gene (Fig. 5A). Although we used an engineered strain to demonstrate this effect (in‐frame *orbJ* deletion), we suggest that the same effects would arise for many types of natural mutations. Specifically, insertions or deletions that lead to frameshifts, or mutations introducing a stop codon in the upstream synthesis genes could all detrimentally affect the downstream receptor gene. In support of our argument, Sokol et al. (1999) showed that ornibactin synthesis mutants in *B. cenocepacia* K56‐2, created through transposon insertions, were compromised in ornibactin uptake. Although the genetic architecture of the ornibactin locus was unknown at the time, this study now retrospectively indicates that insertions lead to the same phenotypes as reported here. Moreover, studies on evolved siderophore nonproducers in *P. aeruginosa* revealed that mutations almost exclusively occurred in regulators of siderophore synthesis (Andersen et al. 2015; Kümmerli et al. 2015), which in our case would lead to the silencing of the entire operon, including the receptor gene. However, it is important to note that mutations in regulators can also lead to mutants with reduced siderophore investment levels (Andersen et al. 2015; Kümmerli et al. 2015). Whether or not regulatory linkage can hinder the invasion of such mutants would depend on how much siderophore synthesis genes are downregulated (saving costs) relative to the reduction of receptor gene expression (ensuring benefits) (Ghoul et al. 2014). Finally, only nonsynonymous SNPs leading to an amino acid substitution would probably not affect downstream receptor expression. However, such mutations would most likely also not lead to the abrogation of ornibactin production and thus not turn producers into potential cheaters. Altogether these considerations show that the discovered mechanism likely confers a robust way to prevent the spread of ornibactin null mutants as cheaters.

If the linkage of siderophore synthesis and receptor genes within the same operon is such a powerful mechanism to prevent cheating, why is it then not ubiquitous across siderophore systems? For instance, in the case of pyochelin (*B. cenocepacia* and *P. aeruginosa*) and pyoverdine, the regulation of siderophore and receptor synthesis are partly decoupled (Visca et al. 2007; Youard et al. 2011; Butt and Thomas 2017). One possible explanation is that there is a trade‐off between the prevention of cheating and the flexibility that can be obtained from independent receptor regulation. Flexible receptor regulation could confer an advantage when bacteria arrive in environments where there is already a pool of secreted siderophores available. In this scenario it was shown for *P. aeruginosa* that bacteria rather rely on pyoverdine recycling than on de novo production to satisfy their need for iron (Imperi et al. 2009; Kümmerli and Brown 2010). This economic mechanism, which can only work if cells are able to selectively upregulate receptor synthesis, also helps siderophore producers to be competitive against cheaters (Kümmerli and Brown 2010). This indicates that natural selection might offer multiple solutions to cope with cheating, and regulatory linkage is only one of them.

Although we have shown that the genetic architecture of a single trait can constrain cheating, other studies have revealed mechanisms against cheating that are based on the regulatory linkage of multiple traits (Foster et al. 2004; Jousset et al. 2009; Dandekar et al. 2012; Ross‐Gillespie et al. 2015; Wang et al. 2015; Majerczyk et al. 2016; Özkaya et al. 2018). For instance, Dandekar et al. (2012) showed that mutants deficient in the LasIR quorum‐sensing (QS) system in *P. aeruginosa* could cheat on cooperative protease production, but exhibited metabolic insufficiencies in certain media, because this QS system also controls the expression of enzymes required for nutrient degradation. Although this pleiotropic effect prevented cheating in their study system, the view that regulatory linkage between traits has specifically evolved for the purpose of stabilizing cooperation has recently been challenged (Dos Santos et al. 2018). The argument is that the evolutionary causality might have been misinterpreted, such that cooperation itself selects for pleiotropy and not the other way round. Put simply, if a regulatory element like a cooperative QS system is in place, it makes economic sense to recruit more than one trait under this regulon, leading to linkage. Moreover, dos Santos et al. (2018) argued that genetic linkage can break and therefore does not reflect a sustainable mechanism to maintain cooperation (see also Wechsler et al. 2019). We believe that our system is different from the ones considered above, because the synthesis and uptake of siderophores are parts of the same trait, and the genetic architecture thus involves some level of linkage and physical proximity in the genome (Fig. 4A) (Visca et al. 2007; Youard et al. 2011; Butt and Thomas 2017). However, the fact that linkage can break over evolutionary time scales persists even for our system. Indeed, a comparative analysis on the variation in the ornibactin locus architecture across different *Burkholderia* species reveals that the genetic linkage reported for *B. cenocepacia* is at least partly broken in two species (*B. cepacia* and *B. paludis*) belonging to the same phylogenetic clade, but also in certain species on neighbouring phylogenetic branches (members of the *B. pseudomallei* group, *B. phytofirmans*, *B. xenovorans*) (Butt and Thomas 2017). Moreover, for one species (*B. paludis*) with broken linkage, we know that it can take up ornibactin without producing it (Butt and Thomas 2017). These considerations indicate that the genetic architecture of social traits can evolve (Dos Santos et al. 2018), and linkage might only offer a temporary solution to withstand cheating.

We now turn to pyochelin of *B. cenocepacia* and ask why the patterns of successful cheating were so different from the ones reported for pyoverdine in *P. aeruginosa* (Fig. 3). We propose that differences in the molecular properties of the two siderophores might explain some of the observed variation (Fig. 3). Specifically, pyochelin is smaller, more diffusible and cheaper to produce compared to pyoverdine (Cornelis 2010; Dumas et al. 2013). The relatively low diffusivity of pyoverdine could explain why the nonproducers’ access to this siderophore is increased in shaken cultures and at high cell density. Conversely, the higher diffusivity of pyochelin could already lead to a more homogenous distribution of this siderophore across cells under static and low‐density conditions, and thus cancel shaking and density effects (Fig. 3E–H). Meanwhile, our observation that pyochelin cheaters perform best when common (Fig. 3K) could be explained by the relatively low pyochelin production costs. When nonproducers are rare, producers can afford losing a low number of cheap molecules without large fitness costs. Conversely, when nonproducers are common, the burden to producers is expected to increase, because most pyochelin molecules will be lost to nonproducers due to their high diffusivity. The scenario seems different for pyoverdine, where high production costs already impose significant fitness losses at low cheater frequencies, whereas lower diffusivity constrains the cheaters’ access to the siderophore at high frequency (Ross‐Gillespie et al. 2007).

In summary, we have established a new system to study siderophore‐meditated social interactions in bacteria. Our experiments revealed yet unknown dynamics between cooperative producers and exploitative cheaters and identified a novel mechanism of how cooperators can become resistant to cheating. Although our study helps to obtain a more nuanced picture on the sociobiology of siderophores, it also highlights that there is likely an enormous diversity of social interactions out there in nature. By focusing on model organisms such as *P. aeruginosa*, we might have so far only looked at the tip of the iceberg.

Associate Editor: K. Lythgoe

## Supporting information


**Table S1**. The plasmid donor strains used in conjugations for tagging H111∆*orbJ*∆*pchAB* with *mcherry*.
**Table S2**. The qPCR primers used in the study.
**Figure S1**. Growth of *B. cenocepacia* strains across a range of iron availabilities.
**Figure S2**. Examples of flow‐cytometry scatter plots from competition experiments between the siderophore nonproducer and siderophore producers.
**Figure S3**. Monoculture growth and competition between the double mutant (H111∆*orbJ*∆*pchAB*) and the *orbA* overexpresser (H111∆*orbJ*∆*pchAB:orbA*).
**Figure S4**. Control experiments feeding supernatants from siderophore producers to the nonproducer H111∆*orbJ*∆*pchAB* in iron rich medium.
**Figure S5**. Control experiments feeding supernatants from siderophore producers to the nonproducer H111∆*orbJ*∆*pchAB:orbA* overexpressing the ornibactin receptor gene from a plasmid.
