# Supplementary material for: Genetic architecture constrains exploitation of siderophore cooperation in the bacterium Burkholderia cenocepacia
Source: Evol Lett. 2019 Oct 2;3(6):610–22. doi: 10.1002/evl3.144 (PMC6906993; doi:10.1002/evl3.144)
Supplement: Supplementary file 1 — Table S1. The plasmid donor strains used in conjugations for tagging H111∆orbJ∆pchAB with mcherry. Table S2. The qPCR primers used in the study. Figure S1. Growth of B. cenocepacia strains across a range of iron availabilities. Figure S2. Examples of flow‐cytometry scatter plots from competition experiments between the siderophore nonproducer and siderophore producers. Figure S3. Monoculture growth and competition between the double mutant (H111∆orbJ∆pchAB) and the orbA overexpresser (H111∆orbJ∆pchAB:orbA). Figure S4. Control experiments feeding supernatants from siderophore producers to the nonproducer H111∆orbJ∆pchAB in iron rich medium. Figure S5. Control experiments feeding supernatants from siderophore producers to the nonproducer H111∆orbJ∆pchAB:orbA overexpressing the ornibactin receptor gene from a plasmid. [file EVL3-3-610-s001.doc]

**Genetic architecture constrains exploitation of siderophore cooperation in the bacterium *Burkholderia cenocepacia***

Santosh Sathe, Anugraha Mathew, Kirsty Agnoli, Leo Eberl and Rolf Kümmerli

**Supplementary Information**

This file contains:

- 2 supplementary tables

- 5 supplementary figures

- supplementary references

**Supplementary tables**

**Table S1. The plasmid donor strains used in conjugations for tagging H111∆*orbJ*∆*pchAB* with *mcherry*.**

| **Strain** | **Phenotype** | **Source** |
| --- | --- | --- |
| *E. coli* S17-1 λpir miniTn7-Ptac-mCherry | The strain carries conjugation elements and mini-Tn7 plasmid with *mcherry* under constitutive promoter. | Rolf Kümmerli's strain collection maintained at the University of Zurich, Switzerland. |
| *E. coli* S17-1 λ pir pUX-BF13 | The strain carries conjugation elements and the conjugation helper plasmid. |

**Table S2. The qPCR primers used in the study.**

| **Primer** | **Sequence (5´-3´)** | **Source** |
| --- | --- | --- |
| Ornibactin synthesis (*orbI*-forward) | TGAATCTGCGGCTCGACAC | Microsynth, Switzerland |
| Ornibactin synthesis (*orbI*-reverse) | CAGTGTGCGGCGATGTGATA |
| Ornibactin receptor (*orbA*-forward) | ACTACAGCCGCTTCGACATC |
| Ornibactin receptor (*orbA*-reverse) | CATCGTGACGGGCGTATACA |
| Housekeeping gene (*recA*-forward) | GCGATCTTCGACATCCTGTA |
| Housekeeping gene (*recA*-reverse) | TTCTCGCCGTTGTAGCTGTA |
| H111 genomic DNA (*ntrC*-forward) | ACAAGGCGGTCGAGTTGAT |
| H111 genomic DNA (*ntrC*-reverse) | ATAGAACTGCCCGTCCGACA |

**Supplementary figures**


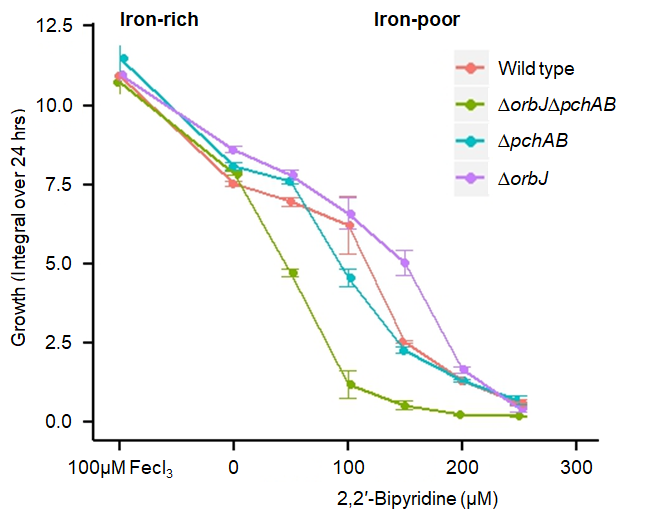


**Figure S1. Growth of *B. cenocepacia* strains across a range of iron availabilities.** *B. cenocepacia* wild type H111 and the three siderophore mutants H111∆*orbJ*∆*pchAB* (producing no siderophores), H111∆*pchAB* (producing ornibactin), and H111∆*orbJ* (producing pyochelin) were either grown in iron-rich CAA medium supplemented with 100 μM FeCl_3_ or in iron-poor CAA media (varying 2, 2’-Bipyridine concentration from 0, 50, 100, 150, 200 to 250 μM). All the strains were grown at 37°C and OD 600 nm was monitored every 15 min for 24 hours. The growth curves were analysed in R using the *grofit* package (Kahm et al. 2010). Because growth trajectories differed fundamentally across conditions, we used spline curve fits and extracted the integral (area under the curve) as growth parameter for comparison. All the strains grew equally well in CAA medium supplemented with 100 μM FeCl_3_ (iron-rich). Iron depletion (upon the addition of bipyridine) significantly reduced growth of all strains, but most severely for the siderophore non-producer (H111∆*orbJ*∆*pchAB*). These results show that siderophores are important for growth.


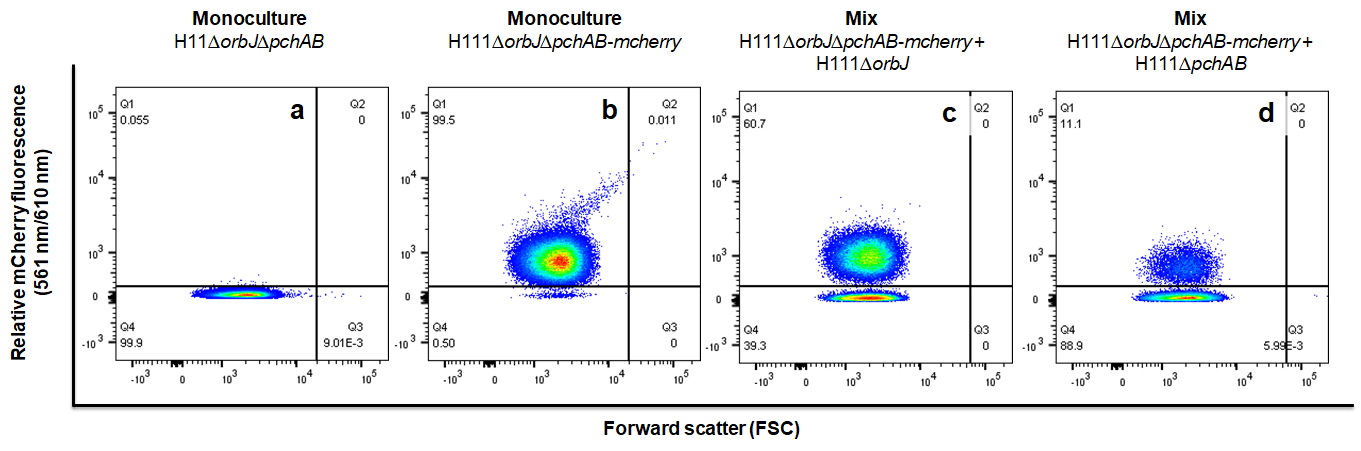


**Figure S2. Examples of flow-cytometry scatter plots from competition experiments between the siderophore non-producer and siderophore producers.** The siderophore double mutant, chromosomally tagged with a constitutively expressed mCherry marker (H111∆*orbJ*∆*pchAB*-*mcherry*) was co-cultured with each of the three siderophore producers (H111 wildtype, H111∆*pchAB,* H111∆*orbJ*). With the flow cytometer, we collected approximately 100,000 events from both mono-cultures and mixed cultures before and after a 24-hours competition period. We then plotted the size of cells (forward scatter, FSC) against the mCherry fluorescence to count cells of both types. (a) A monoculture of the untagged H111∆*orbJ*∆*pchAB* strain does not show mCherry fluorescence. This control enabled as to quantify the background fluorescence of cells. (b) A monoculture of the tagged H111∆*orbJ*∆*pchAB-mcherry* strain show relatively strong mCherry fluorescence, with 99.5 % of all cells considered as mCherry positive. (c) In a 50:50 mix of H111∆*orbJ*∆*pchAB-mcherry* and the pyochelin producer (H111∆*orbJ*) the cells of the two strains can be unambiguously distinguished and their final ratio (60.7:39.3) can be recorded. In this scenario the siderophore non-producer won the competition and acted as a cheater. (d) The same procedure was applied to a 50:50 mix of H111∆*orbJ*∆*pchAB-mcherry* and the ornibactin producer (H111∆*pchAB*). Here, flow cytometry counts revealed a 11.1:88.9 end ratio, showing that the siderophore non-producer clearly lost the competition and could not act as a cheater.


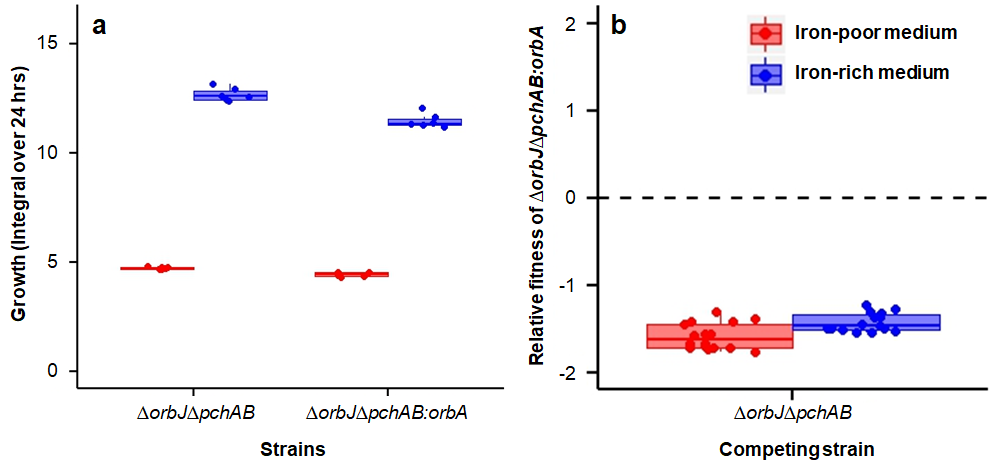


**Figure S3. Monoculture growth and competition between the double mutant (H111∆*orbJ*∆*pchAB*) and the *orbA* overexpresser (H111∆*orbJ*∆*pchAB:orbA*).** (a) The siderophore double mutants H111∆*orbJ*∆*pchAB* and H111∆*orbJ*∆*pchAB* containing a plasmid from which *orbA* is overexpressed were grown in iron-rich and iron-poor CAA media. Growth at OD 600 nm was monitored every 15 min for 24 hours. We extracted the growth integral and compared it between strains. H111∆*orbJ*∆*pchAB:orbA* grew significantly worse than H111∆*orbJ*∆*pchAB*, both in iron-rich (*t*-test: *t*_9.9_, *p* < 0.0001) and iron-poor (*t*_8.2_ = 6.49, *p* = 0.0002) medium. This shows that plasmid carriage has a fitness cost. (b) Costs were confirmed in direct competition assays between the two strains where H111∆*orbJ*∆*pchAB:orbA* significantly lost against H111∆*orbJ*∆*pchAB* (relative fitness lower than zero) both in iron-rich (*t*_14_ = -52.13, *p* < 0.0001) and iron-poor (*t*_15_ = -42.69, *p* < 0.0001) medium. Competitions were performed under static and shaken conditions, but were combined in the plot because the relative fitness values did not differ between the two conditions.


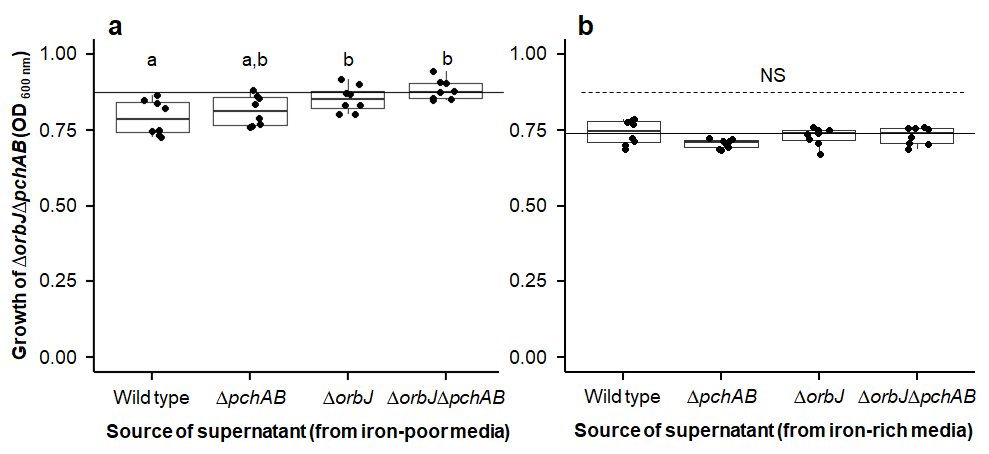


**Figure S4. Control experiments feeding supernatants from siderophore producers to the non-producer H111∆*orbJ*∆*pchAB* in iron rich medium.** The siderophore non-producer H111∆*orbJ*∆*pchAB* was grown in iron-rich medium for 24 hours, supplemented with supernatants collected from all four strains used in our study (H111 wildtype, H111∆*pchAB,* H111∆*orbJ*, H111∆*orbJ*∆*pchAB*). (a) When supernatants from iron-poor medium were supplemented H111∆*orbJ*∆*pchAB* grew slightly better in its own supernatant and the supernatant of H111∆*orbJ* than in the wild type supernatant (*F*_3,28_ = 5.98; *p* = 0.0027). (b) When supernatants from iron-rich medium were supplemented, there was no significant difference between treatments (*F*_3, 28_ = 2.22; *p* = 0.1078). Different letters above the boxplots indicate statistically significant differences between treatments.


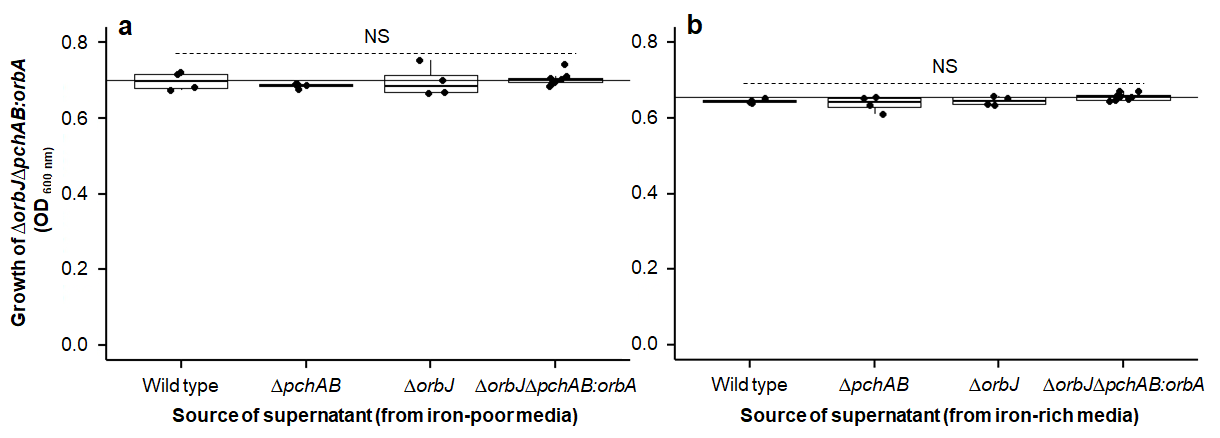


**Figure S5. Control experiments feeding supernatants from siderophore producers to the non-producer H111∆*orbJ*∆*pchAB:orbA* overexpressing the ornibactin receptor gene from a plasmid.** The siderophore non-producer H111∆*orbJ*∆*pchAB:orbA* was grown in iron-rich medium for 24 hours, supplemented with supernatants from H111 wildtype, H111∆*pchAB,* H111∆*orbJ*, and itself. (a) When supernatants from iron-poor medium were supplemented, there was no significant difference between treatments (*F*_3,16_ = 0.56, p = 0.6473). (b) When supernatants from iron-rich medium were supplemented, there was also no significant difference between treatments (*F*_3,16_ = 2.32, *p* = 0.1134).

**Supplementary citation**

1. Kahm, M., Hasenbrink, G., Lichtenberg-Fraté, H., Ludwig, J. and Kschischo, M. 2010. grofit: fitting biological growth curves with R. J. Stat. Softw. 33.
